# Optimization of the Ball Milling Process for Producing Superfine Green Tea Powder: An Analytic Hierarchy Process–Fuzzy Comprehensive Evaluation Approach

**DOI:** 10.3390/foods14071283

**Published:** 2025-04-07

**Authors:** Yangpujia Zhou, Guohao Liu, Tao Zhou, Sui Ni

**Affiliations:** School of Marine Sciences, Ningbo University, Ningbo 315211, China; 2311130071@nbu.edu.cn (Y.Z.); 2211420035@nbu.edu.cn (G.L.); 2311110194@nbu.edu.cn (T.Z.)

**Keywords:** green tea, superfine green tea powder (SGTP), the ball milling method, the AHP–fuzzy comprehensive evaluation approach, the response surface methodology (RSM)

## Abstract

In this study, the ball milling method was used to produce superfine green tea powder (SGTP). We used the contents of chlorophyll, caffeine, tea polyphenols, and total free amino acids as indicators and combined the analytic hierarchy process (AHP) and fuzzy comprehensive evaluation to establish an AHP–fuzzy comprehensive evaluation approach applicable to SGTP. The production process of SGTP was optimized using the response surface methodology (RSM). The results showed that the three factors of grinding time, rotation speed, and ball-to-material ratio had significant effects on the content of the main components of the tea powder, and the order of the effects was as follows: ball-to-material ratio > grinding time > rotation speed. The optimal grinding time, rotation speed, and ball-to-material ratio for the preparation of SGTP were 5.85 h, 397 r/min, and 9.2:1, respectively. We also found that, compared with green tea powder made with the traditional crushing method, the SGTP prepared under these conditions possessed strong advantages in terms of particle size, the content and dissolution of major components, and antioxidant capacity. In this study, the optimization of the production process of tea powder is initially discussed, and then, a new evaluation method for tea powder is proposed, providing technical support for improving the quality of green tea powder. The AHP–fuzzy comprehensive evaluation approach, by quantifying qualitative assessments, significantly refined our optimization process, enabling a more precise determination of optimal SGTP production parameters.

## 1. Introduction

Green tea is a non-fermented tea made from new buds of the *Camellia* plant through the processes of withering, cooling, kneading, and drying. It is one of the most important types of tea worldwide. Research shows that green tea contains a variety of beneficial components for the human body, including polyphenols, vitamins, proteins, amino acids, lipids, polysaccharides, and mineral elements. Green tea also has antioxidant, anti-inflammatory, calming, and blood pressure-lowering properties; promotes metabolism; and prevents cardiovascular diseases [1,2,3,4,5].

Superfine grinding technology is a novel technique that employs mechanical or fluid power to break down objects into powders up to particle sizes at the micron, submicron, and nanometer levels [6]. This process modifies the structure and specific surface area of the materials, leading to new advantageous properties that the raw materials did not possess. Currently, the main methods of superfine grinding include jet milling [7], vibration milling [8], and ball milling [9].

Superfine green tea powder (SGTP) is green tea powder treated with specific technologies, as raw material is ground into powder with a particle size between 1 nm and 100 μm using modern superfine grinding technology [10,11]. Apart from being consumed directly, SGTP is a high-quality additive in various food products used to impart natural green color and tea flavor; it finds applications in bread, ice cream, milk, and other food items. Compared with green tea extract, SGTP retains the nutritional components of green tea more effectively. Moreover, it is better suited for producing various foods and beverages with green tea flavors. Therefore, it has gained widespread application in the field of food processing [12].

The analytic hierarchy process (AHP) is a multi-level decision analysis method proposed by Saaty. This method calculates the weights of individual indicators by constructing matrixes for pairwise comparisons. It is widely used to assign weight values to various indicators in a multi-criteria evaluation system [13,14]. Being an extension of AHP, the AHP–fuzzy comprehensive evaluation approach determines weights through AHP and makes evaluations using fuzzy methods. It effectively converts qualitative evaluations into quantitative evaluations, enabling comprehensive assessments [15]. The AHP–fuzzy comprehensive evaluation approach is widely used in fields such as risk assessment [16], solution selection [17], product design [18], and process optimization [19].

Response surface methodology (RSM) is a statistical method that utilizes an appropriate experimental design and fits a quadratic regression equation to establish the functional relationship between factors and response values. By analyzing the regression equation, optimal process parameters can be determined [20]. This method is widely used in food formulation optimization [21,22].

Chlorophyll is the main pigment in green tea, and its content is one of the most important indexes for judging the quality of green tea powder [23]. Caffeine is the main bitter substance in tea [24]; generally, the caffeine content in tea ranges from 2% to 4% [25]. Moderate amounts of caffeine can complex with tea polyphenols, amino acids, and other components to synthesize refreshing substances. High-quality green tea powder should have a refreshing taste with less bitterness, so slightly lower caffeine content is beneficial to the quality of SGTP. Tea polyphenols are the main antioxidant components of tea, with the effect of scavenging reactive oxygen radicals and inhibiting lipid peroxidation [26,27]. Higher tea polyphenols content is favorable to the quality of tea powder. Free amino acids, being important components, contribute to the fresh taste of tea and provide a refreshing mouthfeel. Free amino acids positively determine SGTP quality [28]. Chlorophyll, caffeine, tea polyphenols, and free amino acids are important components of green tea, and their contents affect the quality of green tea powder. The contents of these components have been used as parts of the indexes for judging the quality of tea powder in previous studies [29,30,31,32,33].

Currently, there is extensive research on the production processes of green tea, black tea, and oolong tea in the industry [34,35,36,37,38,39]. Despite the known benefits of SGTP and the potential of superfine grinding technology, few studies have been conducted on the SGTP production process compared to green tea, black tea and oolong tea, and even fewer on optimizing the ball milling process specifically for SGTP. This study seeks to fill this gap by identifying the optimal ball milling conditions—grinding time, rotation speed, and ball-to-material ratio—that maximize the health-promoting components of SGTP. By doing so, we aim to provide a scientific basis for enhancing the quality and health benefits of superfine green tea powder, aligning with the growing consumer demand for functional foods that support health and well-being.

This study utilized ‘Longjing 43’ green tea as the raw material and employed the ball milling method to produce SGTP. The chlorophyll, caffeine, tea polyphenols (TPs), and total free amino acids (TFAA) contents were employed as indicators to investigate the effects of variables (grinding time, rotation speed, and ball-to-material ratio) on the nutrient composition of SGTP. An AHP–fuzzy comprehensive evaluation system applicable to SGTP was established by combining the AHP with fuzzy comprehensive evaluation. The SGTP production process was optimized using the RSM. In addition, we carried out particle size analysis and morphological observation for the SGTP prepared under optimal process conditions and analyzed its main component contents, dissolution characteristics, and antioxidant capacity. The results provide technical support for improving the quality of green tea powder and screening the optimal parameters of the three processes (grinding time, rotation speed, and ball-to-material ratio) in the ball milling method, and they also provide a reference for the application of superfine grinding technology in food processing.

## 2. Materials and Methods

### 2.1. Materials and Chemicals

‘Longjing 43’ green tea was purchased in August 2022 from Yuyao Zhu Tea Factory (Ningbo, China). Fresh tea shoots, consisting of two leaves and a bud, were plucked by hand from the tea plantation of Yuyao Zhu Tea Factory (Ningbo, China) in May 2022. The feedstock was first steamed and dried and then stored in a refrigerated room.

Ethanol absolute, acetone, methanol, disodium hydrogen phosphate dodecahydrate, potassium dihydrogen phosphate, chloride dihydrate, L-theanine, folin phenol, sodium carbonate, gallic acid, magnesium oxide, ferrous sulfate, and potassium sodium tartrate tetrahydrate were analytical reagents (ARs) purchased from Sinopharm Chemical Reagent Co., Ltd. (Shanghai, China). Ninhydrin (AR) was purchased from Shanghai Titan Scientific Co., Ltd. (Shanghai, China). The caffeine standard (LC) was purchased from Tanmo Quality Inspection Technology Co., Ltd. (Changzhou, China).

### 2.2. Sample Preparation

In the first phase, ‘Longjing 43’ green tea (LJ43) was subjected to primary grinding using a stone mill (Quanzhou Shishanhai Co., Ltd., Quanzhou, China), followed by sieving through a 450 μm sieve (Shaoxing Shangyu Shengchao Instrument Equipment Co., Ltd., Shaoxing, China). The next step was grinding in an XQM-2 vertical planetary ball mill (Changsha Tianchuang Powder Technology Co., Ltd., Changsha, China). Stainless steel grinding balls with a diameter of 5–15 mm were used. The grinding process operated for 10 min in the forward direction, followed by 10 min in the reverse direction, with a 1 min resting time in between. This sequence was repeated several times to finally obtain SGTP samples. This marked the end of the first phase.

The second phase included analyzing the principal components (chlorophyll, caffeine, polyphenols, and amino acids), as well as scanning electron microscopy (SEM) analysis (size and morphology). During this phase, the ground material passed through a stone mill and a high-speed pulverizer (Dongguan Fangtai Electric Appliance Co., Ltd., Dongguan, China). The LJ43 was placed in a high-speed pulverizer; crushed for 30 s to obtain ordinary green tea powder (OGTP); ground in a stone mill; and passed through a 450 μm sieve to obtain coarse green tea powder (CGTP) without superfine grinding. Then, the CGTP was superfine ground according to the optimal process obtained from the first phase to produce BSGTP; the LJ43 was not subjected to any treatment as a control.

### 2.3. Determination of Major SGTP Component Contents

The caffeine content was determined referring to the national standard, GB/T 8312-2013 [40], and caffeine (99.8%, LC) was used as the standard. Standard curve: y = 1465.9x − 1233.1 (R^2^ = 0.9998). The instruments used in the experiment were an Acquity UPLC H-Class HPLC (Waters Corporation, Milford, MA, USA) and an Athena C18-WP column (CNW Technologies GmbH, Dusseldorf, Germany). Chromatographic conditions: UV detection wavelength: 280 nm; mobile phase: mix 600 mL of methanol with 1400 mL of distilled water and filter through a 0.45 μm membrane; flow rate: 1.0 mL/min; column temperature: 40 °C; injection volume: 10 μL.

The chlorophyll content was determined referring to the agricultural industry standard, NY/T 3082-2017 [41]. The TPs content was determined referring to the national standard, GB/T 8313-2018 [42]. The TFAA content was determined referring to the national standard, GB/T 8314-2013 [43]. The instrument used in the experiment was a T6 New Century UV-Vis Spectrophotometer (Beijing Puxi General Instrument Co., Ltd., Beijing, China). The chlorophyll content was determined using UV spectrophotometry. An ethanol–acetone (1:1, *v*/*v*) mixture was used as the extractant, and the detection wavelengths were set at 645 nm and 663 nm. The TPs content was determined according to the UV spectrophotometric method using gallic acid as calibrant. The detection wavelength was set at 765 nm. The chlorophyll content was determined using UV spectrophotometry with L-theanine as the standard. The detection wavelength was set at 570 nm.

### 2.4. AHP–Fuzzy Comprehensive Evaluation of SGTP

Traditional evaluation methods are inherently subjective due to their susceptibility to the personal preferences of sensory judges. To address this limitation, this study establishes an AHP–fuzzy comprehensive evaluation system that combines AHP with traditional evaluation methods for SGTP. Using the AHP, the weights of various indicators can be determined. Next, the multilevel fuzzy evaluation approach was used to score and evaluate the SGTP indicators. Lastly, the SGTP quality was assessed based on the sample’s comprehensive scores from both methods.

#### 2.4.1. AHP to Determine the Weight of Each Indicator

A 4th-order judgment matrix was established using four evaluation indicators: chlorophyll content, caffeine content, TPs content, and TFAA content. Ten experts from the fields of botany, tea studies, food engineering, and biology were invited as reviewers. The matrix indicators were discussed and scored on a scale of 1–5 based on their relative importance. For instance, if A was considered significantly more important than B and assigned a score of 3, then B would receive a score of 1/3, equivalent to 0.33. The relative weights of the evaluation indicators were then calculated, and the consistency of the results was verified. The scoring method for the judgment matrix is detailed in Table 1.

#### 2.4.2. Fuzzy Comprehensive Evaluation of SGTP

After using the AHP to determine the weight value of each index, 10 experts from the botany, tea studies, food engineering, and biology fields were invited as reviewers to score the SGTP samples obtained after different grinding processes. The final results were taken as the average scores provided by the 10 reviewers. The specific scoring method is shown in Table 2.

### 2.5. Single-Factor Experiment

The effects of grinding time, rotation speed, and ball-to-material ratio (mass ratio) on the chlorophyll, caffeine, TPs, and TFAA contents in SGTP were investigated through single-factor experiments by controlling the variables.

The CGTP was placed in an XQM-2 vertical planetary ball mill for superfine grinding. The default grinding conditions were a grinding time of 7 h, rotation speed of 400 r/min, and ball-to-material ratio of 10:1. After obtaining the SGTP samples under different conditions, the samples were tested for the chlorophyll, caffeine, TPs, and TFAA contents. The samples in each group were scored according to the method described in Section 2.4, and then, the optimal single-factor conditions for the preparation of SGTP were determined using the AHP–fuzzy comprehensive evaluation score. The method is detailed in Table 3.

### 2.6. Response Surface Optimization Experiment

Based on the previous single-factor experiments, a Box–Behnken model was employed to design a three-factor, three-level response surface optimization experiment (Table 4). This experiment aimed to determine the optimal grinding technique for producing SGTP.

### 2.7. Particle Size Analysis of BSGTP

An appropriate amount of sample was weighed and dispersed using distilled water as a dispersant. After the sample and distilled water were mixed thoroughly, the sample was analyzed for particle size. The instrument used in the experiment was a Mastersizer 3000 laser particle size analyzer (Malvern Panalytical Ltd., Malvern, UK). The instrument measurement parameters were set as follows: ultrasonic intensity, 10 W/cm^2^; refractive index, 1.52; upper and lower lines, 10–20%; rotation speed, 2500 r/min. The D_10_ (diameter of 10% of particles below this value), D_50_ (median particle size, diameter of 50% of particles below this value), D_90_ (diameter of 90% of particles below this value), D_(3, 2)_ (surface weighed mean diameter), D_(4, 3)_ (volume-weighted mean diameter), SPAN (distribution width), and specific surface area of the powders were measured under these conditions, and the cell wall breakage ratio (Φ) was calculated using the following equation:

(1) $$\Phi =\left[1\;-\left(1\;-\frac{10}{D_{50}}\right)\right]\;\times \;100\%$$

### 2.8. Morphological Observation of BSGTP

A suitable carrier table size was selected to paste the conductive adhesive, and then, an appropriate amount of sample was dialed onto the conductive adhesive using a dissecting needle. The samples were fixed via gold spraying with an E-1010 ion sputtering system (Hitachi, Ltd., Tokyo, Japan). The microstructural characteristics of the samples were then observed under an S-3400N scanning electron microscope (Hitachi, Ltd., Tokyo, Japan).

### 2.9. Determination of Major BSGTP Component Contents

The TPs content was determined using a spectrophotometric method [44]. Ferrous tartrate solution was used as the color developer, in which Fe^2+^ can form blue-violet compounds with polyphenols. The detection wavelength was set at 540 nm. The instrument used in the experiment was a T6 New Century UV–Vis Spectrophotometer (Beijing Puxi General Instrument Co., Ltd., Beijing, China). The chlorophyll, caffeine, and TFAA contents were determined using the same methods described in Section 2.3.

### 2.10. Dissolution Determination of BSGTP

After weighing 0.3 g of the sample in a 50 mL centrifuge tube, 45 mL of distilled water at 37 °C was added, followed immediately by a 37 °C constant-temperature water bath. The samples were extracted for 0.5 min, 1 min, 3 min, 5 min, 10 min, 20 min, 30 min, and 50 min. After extraction, the test solution was centrifuged at 10,000× *g* for 10 min, and the supernatant was passed through a 0.45 μm filter membrane. The filtrate was transferred into a 50 mL volumetric flask and shaken well after adding distilled water to the scale; then, the sample dissolution solution was obtained. It was used to determine the dissolution of the major components of different samples at 37 °C. The TPs, caffeine, and TFAA contents in the sample dissolution solution were determined using the same methods described in Section 2.9.

### 2.11. Study of Antioxidant Capacity of BSGTP

The total antioxidant capacity of the samples was determined using the FRAP-2-G kit (Suzhou Keming Biotechnology Co., Ltd., Suzhou, China). The DPPH free-radical-scavenging capacity of the samples was determined using the BC4750 kit (Beijing Solarbio Science & Technology Co., Ltd., Beijing, China). The hydroxyl free-radical-scavenging capacity of the samples was determined using the QZQ-2-G kit (Suzhou Keming Biotechnology Co., Ltd., Suzhou, China). The instrument used in the experiment was a T6 New Century UV–Vis Spectrophotometer (Beijing Puxi General Instrument Co., Ltd., Beijing, China).

### 2.12. Statistical Analysis

All experiments were replicated three times, and the final results were averaged. Microsoft Excel 2019 was used for data statistics, SPSS Statistics 25 for data processing, Design Expert 13 for response surface experimental design and the generation of response surface images, and OriginPro 2024 and Adobe Illustrator 2020 for graphing.

## 3. Results and Discussion

### 3.1. Results of AHP for Determining the Weight of Each Indicator

A fourth-order judgment matrix (Table 5) was established using four evaluation indicators: chlorophyll content, caffeine content, TPs content, and TFAA content.

According to Saaty’s method, the fourth-order judgment matrix in Table 5 was analyzed using the sum–product method [14]. The characteristic vectors of the four indicators were 0.490, 0.909, 0.909, and 1.692. The corresponding weight values for caffeine content, chlorophyll content, TPs content, and TFAA content were 12.252%, 22.718%, 22.718%, and 42.312%, respectively. The maximum eigenvalue of the judgment matrix was 4.010. Using the maximum eigenvalue, the consistency index (CI) was calculated to be 0.003. The CI was used for subsequent consistency checks. Please refer to Table 6 for the detailed results of the AHP. The CI was calculated using the following equation:

(2) $$\mathrm{CI}=\frac{\left(\mathrm{maximum}\;\mathrm{eigenvalue}\;-\;n\right)}{n\;-\;1}$$

When applying the AHP method for weight calculation, it is essential to conduct a consistency check analysis to evaluate the consistency of the weight calculation results. This assessment involves computing the consistency ratio (CR) to evaluate the consistency of the judgment matrix. The CR was calculated using the following equation:

(3) $$\mathrm{CR}=\frac{\mathrm{CI}}{\mathrm{RI}}$$

Generally, a lower CR indicates improved consistency, and a CR below 0.1 indicates that the judgment matrix passes the consistency check. The previously calculated CI was 0.003. The random consistency index (RI), obtained from the table of RI values, was 0.890. Therefore, as the calculated CR is 0.004 < 0.01, the judgment matrix established in this study passes the consistency check, indicating scientifically reasonable weights. For the specific results of the consistency check, refer to Table 7.

### 3.2. Results of Single-Factor Experiment

#### 3.2.1. Grinding Time

Table 8 displays the contents of the major components in the SGTP obtained at various grinding times. These results indicate that as the grinding time increases, chlorophyll, caffeine, and TPs in the samples initially rise before declining. The sample ground for 6 h exhibits the highest chlorophyll content at 5.01 mg/g; the sample ground for 7 h shows the highest caffeine content and TPs content at 2.46% and 11.81%, respectively. The TFAA content of the samples shows a gradual decrease. Specifically, the sample ground for 5 h exhibits the highest content at 2.81%, and the sample ground for 9 h shows the lowest content at 2.66%.

The superfine grinding process disrupts the cell walls of tea leaves, leading to the release of chlorophyll, thereby making the tea powder appear greener. Simultaneously, superfine grinding augments the specific surface area of the powder, consequently facilitating the dissolution of substances such as TPs and caffeine [45]. However, when the grinding time exceeds 7 h, the prolonged friction and collision among the grinding jar, grinding balls, and samples generate substantial heat, leading to the thermal degradation of certain chlorophyll and caffeine components within the samples. Moreover, the particle size of the SGTP gradually decreases with the increase in grinding time [29]. Thus, TPs are more likely to be oxidized with a small amount of air inside the grinding jar in an enclosed and high-temperature environment, resulting in a decrease in their content [46]. In addition, free amino acids in the tea powder are released after superfine grinding. After a long time in a sealed and high-temperature jar, free amino acids will undergo Maillard reactions with tea polysaccharides, ketones, polyphenols, and other substances, leading to a decrease in content [47,48]. In general, an extended grinding time promotes the release of intrinsic components within the tea leaves. However, considering the influence of machine-generated heat during practical operations, the grinding time should not be excessively long.

**Figure 1:**
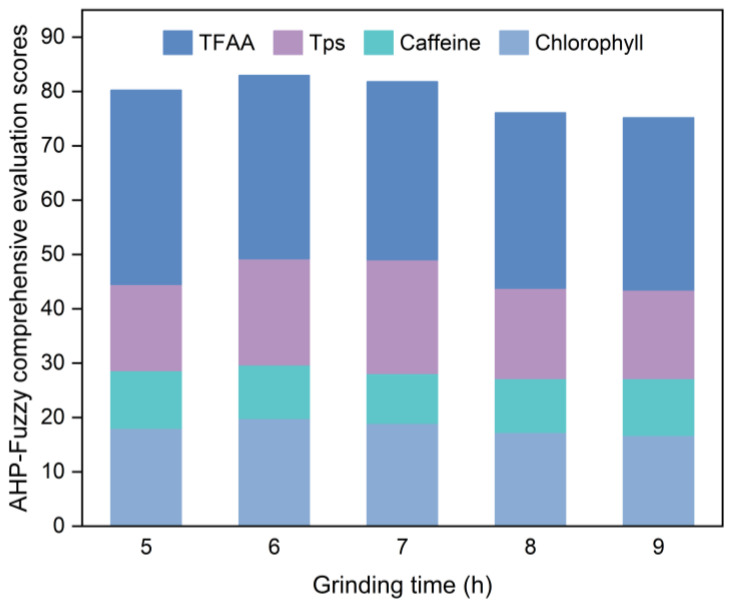
AHP–fuzzy comprehensive evaluation scores for SGTP under different grinding time conditions. Remaining operating conditions: rotation speed of 400 r/min and ball-to-material ratio of 10:1.

Figure 1 illustrates the results of the evaluation conducted following the guidelines outlined in Section 2.4.2 on SGTP samples produced with different grinding times.

Based on Figure 1, the AHP–fuzzy comprehensive evaluation scores for the five sample groups with different grinding times are ranked as follows, from lowest to highest: 9 h < 8 h < 5 h < 7 h < 6 h. The sample with a grinding time of 6 h has the highest AHP—fuzzy comprehensive evaluation score at 82.95. The sample ground for 6 h exhibited the highest chlorophyll content among the five sample groups, measuring 5.01 mg/g. Moreover, its caffeine content (2.41%) is relatively close to the values of the other four sample groups. Furthermore, its TPs content ranked second only to the sample ground for 7 h at 11.51%. It also contained 2.76% TFAA content, ranking second only to the sample ground for 5 h. Consequently, the optimal grinding time was 6 h.

#### 3.2.2. Rotation Speed

Table 9 displays the contents of the major components in the SGTP obtained at various rotation speeds. These results indicate that as the rotation speed accelerates, chlorophyll, caffeine, and TFAA in the samples initially rise before declining. The highest caffeine content in the samples was 2.47% when the rotation speed reached 300 r/min; the highest chlorophyll and TFAA contents in the samples were 4.94 mg/g and 2.72% when the rotation speed reached 400 r/min, respectively. The TPs content of the samples showed a gradual increase. Specifically, the sample exhibited the lowest content at 8.47% when the rotation speed reached 200 r/min and the highest content at 10.96% when the rotation speed reached 600 r/min.

When Chen et al. [49] studied the micronization process (rotation speed and grinding ball size) for okara, they found that the total phenolic content was directly proportional to the rotation speed when 5 mm grinding balls were used. This finding is consistent with the results of the present study. When 1 mm grinding balls were used, the total phenolic content showed an increasing and then decreasing trend with an increasing rotation speed. The total phenolic content of the samples produced at a rotation speed of 750 r/min was lower than that of the samples produced at a rotation speed of 500 r/min. The reason may be that the rotation speed parameter designed by Chen et al. is relatively large. The maximum rotation speed parameter designed in this study was only 600 r/min, so the rotation speed may not have reached the level where the TPs content started to decrease. In addition, in the actual experimental process, with a low rotation speed of 200 r/min, the tea powder gathered at the bottom of the grinding jar, while the grinding balls moved above the tea powder. At the end of the ball milling process, the SGTP was compacted at the bottom of the canister by the grinding balls, resulting in the powder being unable to fully collide with the grinding balls, making the superfine grinding efficiency lower. The color of the SGTP produced when the rotation speed was set at 600 r/min was burnt yellow instead of vibrant green, which was poor in sensory quality. It was hypothesized that the reason for this was that too large a rotation speed resulted in too intense collisions inside the canister during grinding, increasing the temperature inside the canister. Thus, the chlorophyll of the tea powder was thermally decomposed. The changes in the caffeine and TFAA contents showed a similar trend to the changes in chlorophyll content, both increasing and then decreasing. Hence, to ensure the production of high-quality SGTP, a suitable rotation speed should be carefully chosen.

**Figure 2:**
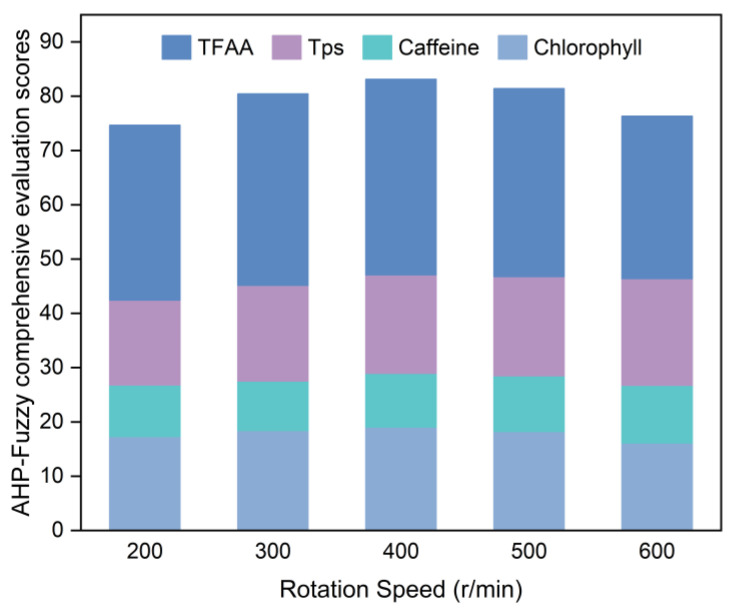
AHP–fuzzy comprehensive evaluation scores for SGTP under different rotation speed conditions. Remaining operating conditions: grinding time of 7 h and ball-to-material ratio of 10:1.

Figure 2 illustrates the results of the evaluation conducted following the guidelines outlined in Section 2.4.2 on SGTP samples produced with different rotation speeds.

Based on Figure 2, the AHP–fuzzy comprehensive evaluation scores for the five sample groups with different rotation speeds are ranked as follows, from lowest to highest: 200 r/min < 600 r/min < 300 r/min < 500 r/min < 400 r/min. The sample at a rotation speed of 400 r/min has the highest AHP–fuzzy comprehensive evaluation score at 83.12. It has the highest chlorophyll and TFAA contents among the five sample groups, with values of 4.94 mg/g and 2.72%, respectively. Moreover, its caffeine and TPs contents were 2.45% and 10.40%, respectively, belonging to the medium level among the five sample groups. Consequently, the optimal rotation speed was 400 r/min.

#### 3.2.3. Ball-to-Material Ratio

Table 10 displays the contents of the major components in the SGTP obtained at various ball-to-material ratios. These results indicate that as the ball-to-material ratio increases, the chlorophyll, TPs, and TFAA in the samples initially rise before declining. The highest chlorophyll content in the samples was 5.23 mg/g when the ball-to-material ratio was 12:1; the highest TPs and TFAA contents in the samples were 11.91% and 2.63% when the ball-to-material ratio was 8:1, respectively. The caffeine content in the samples showed a gradual increase. Specifically, the sample exhibited the lowest content at 2.38% when the ball-to-material ratio was 6:1 and the highest content at 2.48% when the ball-to-material ratio was 14:1.

Numerous studies in the domain of materials science have demonstrated that modifying the ball-to-material ratio during the ball milling process can impact the physical characteristics of the powder obtained, thereby allowing for product optimization through ratio adjustments [40,41,42,43,44,45,46,47,48,49,50,51,52]. In the research on the use of ball milling to produce powders from plants, most scholars have focused more on studying the effect of grinding time on powder quality [53,54,55], disregarding the influence of other conditions. This research demonstrates that the ball-to-material ratio has a significant effect on SGTP quality. The experimental findings demonstrate that an increased ball-to-material ratio yields a finer and more delicate powder, consequently facilitating the release of components (chlorophyll, caffeine, TPs, and TFAA) from the tea powders. However, notably, an excessively high ball-to-material ratio can diminish grinding efficiency and generate excessive heat [56], ultimately compromising the SGTP quality. Hence, it is imperative to carefully determine an appropriate ball-to-material ratio in accordance with the specific circumstances.

**Figure 3:**
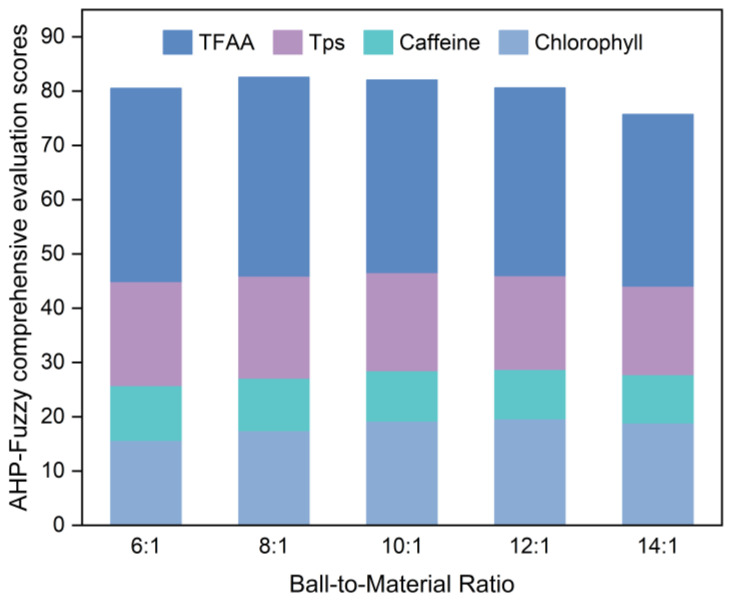
AHP–fuzzy comprehensive evaluation scores for SGTP under different ball-to-material ratio conditions. Remaining operating conditions: grinding time of 7 h and rotation speed of 400 r/min.

Figure 3 illustrates the results of the evaluation conducted following the guidelines outlined in Section 2.4.2 on SGTP samples produced with different ball-to-material ratios.

Based on Figure 3, the AHP–fuzzy comprehensive evaluation scores for the five sample groups with different ball-to-material ratios are ranked as follows, from lowest to highest: 14:1 < 6:1 < 12:1 < 10:1 < 8:1. The sample at a ball-to-material ratio of 8:1 has the highest AHP–fuzzy comprehensive evaluation score at 82.55. The chlorophyll content of the sample at a ball-to-material ratio of 8:1 was 4.77 mg/g, and its caffeine content was the lowest among the five sample groups, measuring 2.41%. In addition, it had the highest TPs and TFAA contents among the five sample groups, with values of 11.91% and 2.63%, respectively. Consequently, the optimal ball-to-material ratio was 8:1.

### 3.3. Optimization of the Optimal Grinding Process for SGTP

#### 3.3.1. Box–Behnken Modeling and Significance Analysis

**Figure 4:**
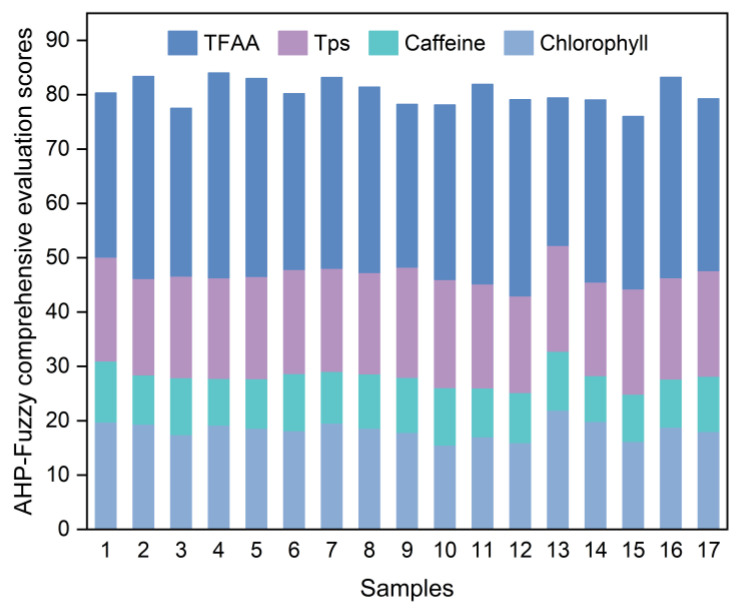
AHP–fuzzy comprehensive evaluation scores for response surface samples.

Based on the results of the single-factor experiments, we selected grinding time (A), rotation speed (B), and ball-to-material ratio (C) as independent variables. The sample’s AHP–fuzzy comprehensive evaluation score (Y) was used as the response value. To optimize the SGTP’s grinding technology, we employed a Box–Behnken model in a three-factor, three-level design, resulting in seventeen groups with five replicate experiments at the center points. The experiments were conducted in a random order. The specific experimental design and results can be seen in Table 11 and Figure 4. The HPLC chromatograms of the caffeine standard and BSGTP are shown in Figure S1.

Table 11 shows that the actual experimental scores for the AHP–fuzzy comprehensive evaluation of the SGTP samples are close to the predicted scores of the model, indicating that it fits the experimental data well and can effectively simulate and predict an actual experimental situation. By analyzing the data in Table 11, the quadratic regression equation for the AHP–fuzzy comprehensive evaluation score (Y) of the SGTP can be obtained as Equation (4). The analysis of variance results are shown in Table 12.

Y = 20.27429A + 0.288451B + 10.08147C − 0.003684AB − 0.581726AC − 0.005067BC − 1.14749A^2^ − 0.000278B^2^ − 0.254261C^2^ − 79.57238 (4)

Note: A is the grinding time (h); B is the rotation speed (r/min); C is the ball-to-material ratio.

**Table 12:** ANOVA for response surface quadratic model.

Source	Sum of Squares	DF	Mean Square	F-Value	*p*-Value	Inference
Model	88.63	9	9.85	31.11	<0.0001	**
A	4.05	1	4.05	12.79	0.0090	**
B	2.77	1	2.77	8.76	0.0211	*
C	10.59	1	10.59	33.45	0.0007	**
AB	0.54	1	0.54	1.72	0.2317	not significant
AC	12.18	1	12.18	38.48	0.0004	**
BC	9.24	1	9.24	29.19	0.0010	**
A^2^	5.54	1	5.54	17.51	0.0041	**
B^2^	32.48	1	32.48	102.60	<0.0001	**
C^2^	22.05	1	22.05	69.65	<0.0001	**
Residual	2.22	7	0.32			
Lack of Fit	1.62	3	0.54	3.61	0.1234	not significant
Pure Error	0.60	4	0.15			
Cor Total	90.85	16				
R^2^ = 0.9756; R^2^_adj_ = 0.9442						

Note: * indicates statistical significance with *p* < 0.05, ** indicates statistical significance with *p* < 0.01.

Table 12 shows that the model has a *p*-value < 0.0001, indicating that it has significant differences. Moreover, the value for the lack-of-fit term is 0.1234 (*p* > 0.05), which is not significant. The R^2^ value is 0.9756, and the adjusted R^2^ is 0.9442, indicating that the model can effectively simulate the relationship between the independent variables and the response value, showing high reliability and validity.

**Figure 5:**
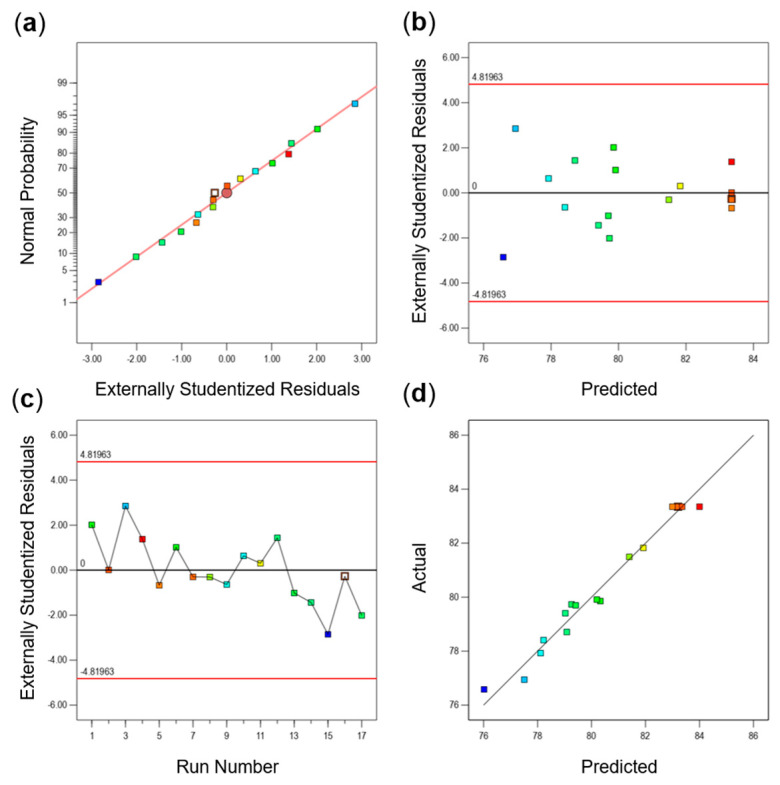
Residual analysis of response surface. (**a**) Probability distribution of externally studentized residuals, (**b**) plot of residuals versus predicted, (**c**) plot of externally studentized residuals versus run number, and (**d**) plot of actual versus predicted values.

As can be seen from Figure 5a, the experimental data points in the figure are basically distributed on a straight line, indicating that the standard deviation deviates less from the actual or predicted value, and the residuals of the model are normally distributed. In Figure 5b, the scattered points are randomly distributed above and below the “0” horizontal axis and do not show a trumpet or funnel shape, indicating that the model fitting effect is better. In Figure 5c, the residual plots are distributed in a random manner and do not show any trend, which indicates that the hypotheses tested are independent. Figure 5d shows that the fitted curves of the actual and predicted points basically coincide with each other, indicating that the model fits well. The above results show that the model is reliable and reasonable.

Among the first-order terms, A and C have extremely significant effects on the response value (*p* < 0.01), and B has a significant effect on the response value (*p* < 0.05). Among the quadratic terms, A^2^, B^2^, and C^2^ all have extremely significant effects on the response value (*p* < 0.01). Based on the F-values for each factor, the order of influence of the three independent variables on the response value is as follows: ball-to-material ratio (F_C_ = 33.45) > grinding time (F_A_ = 12.79) > rotation speed (F_B_ = 8.76).

#### 3.3.2. Interaction Effects of Two Factors

**Figure 6:**
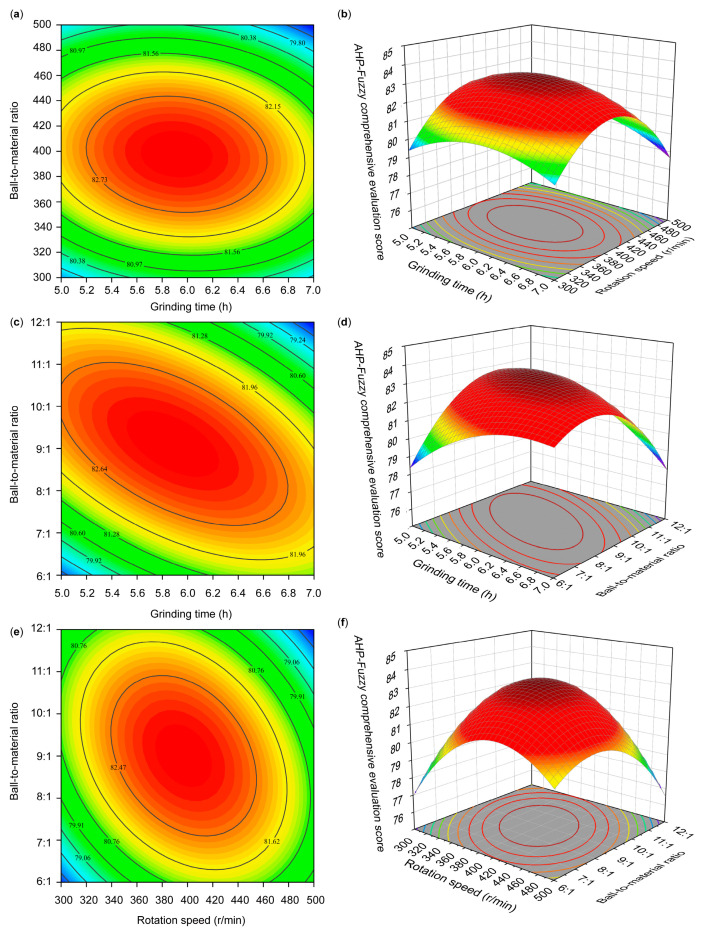
Contour maps and surface maps of AHP–fuzzy comprehensive evaluation scores for two-factor interactions: contour map (**a**) and surface map (**b**) for the interaction between grinding time and rotation speed; contour map (**c**) and surface map (**d**) for the interaction between grinding time and ball-to-material ratio; contour map (**e**) and surface map (**f**) for the interaction between rotation speed and ball-to-material ratio.

The results of the two-factor interactions are shown in Figure 6. Figure 6a,b show that the interaction between grinding time and rotation speed had a non-significant effect (*p* > 0.05) on the AHP–fuzzy comprehensive evaluation scores of the samples. Figure 6c–f indicate that the interaction of grinding time with the ball-to-material ratio and the interaction of rotation speed with the ball-to-material ratio had extremely significant effects (*p* < 0.01) on the AHP–fuzzy comprehensive evaluation scores of the samples. These results are consistent with the analysis of variance results from the response surface optimization experiments shown in Table 12.

#### 3.3.3. Optimal Grinding Process Optimization and Validation of SGPT

Using Design Expert 13, the optimal grinding technology for SGPT was fitted. The results showed that the optimal grinding parameters for SGTP were a grinding time of 5.8687 h, a rotation speed of 396.97 r/min, and a ball-to-material ratio of 9.1515:1. Under these conditions, the sample’s AHP–fuzzy comprehensive evaluation score was 83.3630. The optimal grinding parameters for SGTP were refined to facilitate operational requirements in practical production. The refined process included a grinding time of 5.85 h (equivalent to 351 min), a rotation speed of 397 r/min, and a ball-to-material ratio of 9.2:1. Three validation experiments were conducted under these conditions, and the average AHP–fuzzy comprehensive evaluation score of the final samples was 83.3519, closely matching the predicted values of the model. This demonstrates that the model can effectively simulate real situations and is reliable.

In conclusion, the optimal pulverization technology for SGTP was determined to be a grinding time of 5.85 h, a rotation speed of 397 r/min, and a ball-to-material ratio of 9.2:1.

### 3.4. Particle Size Determination for BSGTP

**Figure 7:**
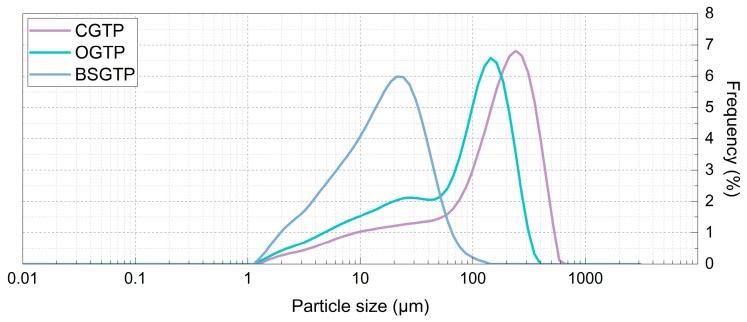
Particle size distribution of three sample groups.

The analysis of the particle size distribution of the three sample groups (Figure 7) shows that almost all of the BSGTP samples were smaller than 100 μm, thus falling within the particle size range of ultrafine powders.

Table 13 shows that the particle sizes of CGTP, OGTP, and BSGTP were mainly distributed between 13 and 381 μm, 8 and 223 μm, and 4 and 44 μm, respectively. The SPANs of the three sample groups are ranked as follows, from lowest to highest: CGTP < BSGTP < OGTP. The D_(3, 2)_ and D_(4, 3)_ of the CGTP were both the largest among the three groups, at 33.3 μm and 183 μm, respectively, while the D_(3, 2)_ and D_(4, 3)_ of the BSGTP were both the smallest, at 9.75 μm and 21.2 μm, respectively.

Among the three sample groups, CGTP had the smallest specific surface area at 180.4 m^2^/kg, and BSGTP had the largest specific surface area at 615.5 m^2^/kg, which was significantly higher than the other two groups. Specific surface area is the total area per unit mass of a material. In general, the smaller the particle size of a material, the larger its specific surface area. An increase in specific surface area can significantly change the solubility of a material [57]. Compared with other grinding methods, superfine grinding can significantly reduce the particle size of a sample, thus increasing its specific surface area. The SGTP can be applied as a food additive to some beverages in the food field, such as milk tea, yogurt, and so on. Therefore, for green tea powder, having a larger specific surface area allows the powder to better combine with water, thus promoting better dissolution in the components of the tea powder.

In addition, among the three sample groups, the cell wall breakage ratios of BSGTP, OGTP, and CGTP were 93.019%, 29.193%, and 16.815%, respectively. The cell wall breakage ratio of the BSGTP was significantly higher than that of the other samples. To a certain extent, a higher cell wall breakage ratio means more green tea cell walls are destroyed, resulting in the increased leaching of amino acids, tea polysaccharides, TPs, and other substances from the cells, improving the flavor and antioxidant capacity of the tea soup.

### 3.5. Results of Morphological Observation of BSGTP

**Figure 8:**
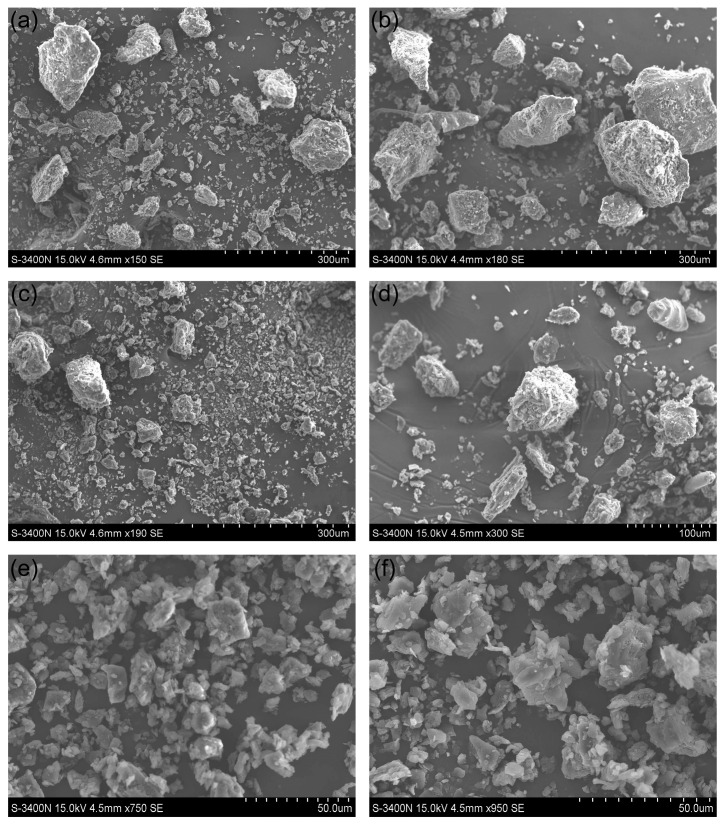
Microscopic appearances of CGTP, OGTP, and BSGTP. (**a**,**b**) are the microscopic appearances of CGTP at 150× and 180× magnifications, respectively. (**c**,**d**) are the microscopic appearances of OGTP at 190× and 300× magnifications, respectively. (**e**,**f**) are the microscopic appearances of BSGTP at 750× and 950× magnifications, respectively.

To further understand the morphological properties of SGTP, the samples were observed using SEM, and differences in shape and size were found between the powders treated with and without superfine grinding. The microscopic appearances of CGTP, OGTP, and BSGTP are shown in Figure 8.

Figure 8a,b show that the CGTP particles were large and irregularly shaped. The distribution of different particle sizes was extremely uneven, and uncrushed primary leaf vein fragments and petiole fragments could be clearly observed. Given Figure 8c,d, the particle size of OGTP is obviously smaller than that of CGTP, but the particle size distribution was also not uniform; there were still some large particles, so we can conclude that the multifunctional pulverizer does not crush tea leaves well. Presumably, the reason is that during the crushing process, smaller particles are clustered at the bottom of the crusher cavity, while larger particles are located in the cavity above; thus, during the operation, the crushing blades at the bottom of the machine cannot make sufficient contact with the particles at the top, resulting in uneven crushing. This may also be because, to prevent overheating, the pulverizer must use a shorter crushing time, making the crushing insufficient. Figure 8e,f show that the BSGTP particles are small and mostly irregular block structures, and the particles with different sizes are uniformly distributed.

These observations are consistent with the particle size measurements, so it is evident that the superfine grinding technique can grind tea leaves well to achieve the desired effect.

### 3.6. Major BSGTP Component Contents

**Figure 9:**
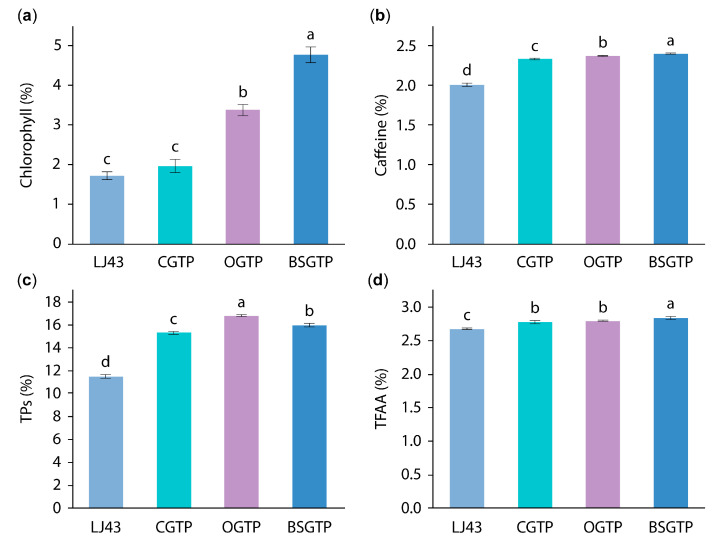
Contents of different major components in four sample groups (*p* < 0.05): (**a**) chlorophyll, (**b**) caffeine, (**c**) TPs, and (**d**) TFAA. Different letters a–d: xxx. Values in a single figure with different letters are significantly different (*p* < 0.05).

The chlorophyll, caffeine, TPs, and TFAA contents of the four sample groups are shown in Figure 9.

Chlorophyll, caffeine, and free amino acids are the main active ingredients in tea and important indicators for evaluating tea quality. Based on Figure 9a,b,d, the chlorophyll, caffeine, and TFAA contents of the four sample groups are ranked as follows, from lowest to highest: LJ43 < CGTP < OGTP < BSGTP. BSGTP was significantly higher than the other three groups. The presumed reason for this is that superfine grinding brings the sample particle size to the micron level, effectively disrupting the cellular structure of the tea leaves (with a 93.019% cell wall breakage ratio for BSGTP), leading to enhanced extractability due to the structural disruption of chlorophyll, caffeine, and free amino acids [30,58].

TPs are important substances that determine the flavor and antioxidant capacity of tea. Based on Figure 9c, the chlorophyll, caffeine, and TFAA contents of the four sample groups are ranked as follows, from lowest to highest: LJ43 < CGTP < BSGTP < OGTP. The TPs content of the BSGTP was significantly lower than that of the OGTP and significantly higher than that of the LJ43 and CGTP. It is hypothesized that the low tea polyphenol content of the BSGTP compared with that of the OGTP is due to the unstable nature of TPs and the fact that catechins, the most abundant substances in TPs, are susceptible to oxidative reactions at high temperatures and under neutral or alkaline conditions [59]. During superfine grinding, the increased specific surface area of the powder makes the contact area of BSGTP with oxygen larger, leading to catechin oxidation. At the same time, a long superfine grinding time will produce more heat, raising the temperature of the canister, which also promotes catechin oxidation, resulting in more losses [30]. In contrast, the crushing time for OGTP was only 30 s. The shorter crushing time retained more TPs in the tea leaves, resulting in low TPs content in BSGTP compared with OGTP.

### 3.7. Dissolution of BSGTP

**Figure 10:**
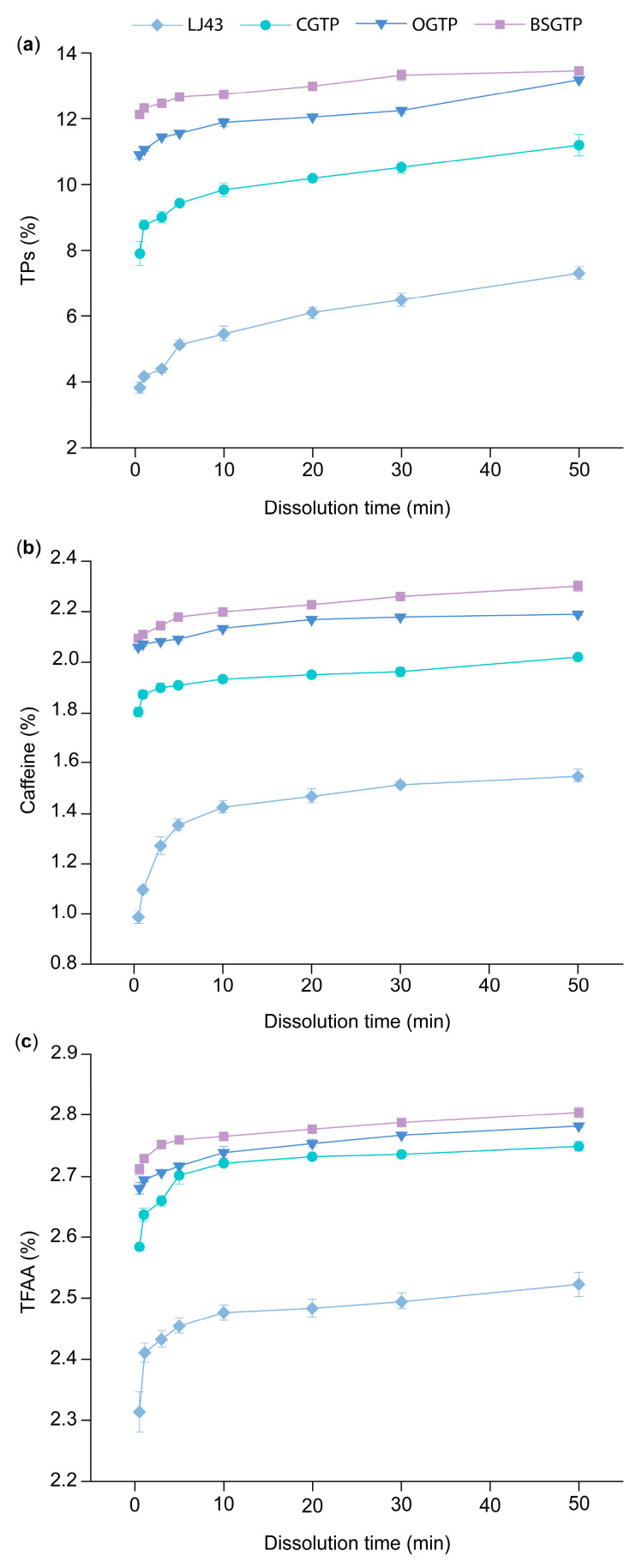
Dissolution of different components in 4 sample groups. (**a**) TPs, (**b**) caffeine, and (**c**) TFAA.

The relationship between the dissolution of different components in the four sample groups and the extraction time is shown in Figure 10.

Figure 10 shows that the dissolution of caffeine, TPs, and free amino acids in all sample groups increased continuously with the prolongation of extraction time, and BSGTP, OGTP, and CGTP were significantly higher than LJ43. Xiao et al. [60] and Li et al. [61] observed a similar phenomenon, so it is believed that the grinding treatment can effectively dissolve the inner contents of tea leaves.

Figure 10a shows that the change in TPs dissolution for BSGTP from the beginning at 0.5 min to the end at 50 min of extraction was only 1.33%, while it was 2.29%, 3.30%, and 3.49% for OGTP, CGTP, and LJ43, respectively. This indicates that BSGTP has a faster TPs dissolution rate, tending to approach the maximum dissolution in a short period of time; thus, more TPs can be dissolved in less time. Jo et al. [62] studied Jerusalem artichoke tea and found that the total phenolic content of ground tea was significantly higher than that of sliced tea, concluding that TPs diffusion was more efficient in the former; i.e., the particle size of the tea leaf affected the extraction efficiency.

Based on Figure 10b, the caffeine dissolution rates of the four groups of samples for each extraction period are ranked as follows, from lowest to highest: LJ43 < CGTP < OGTP < BSGTP. The dissolution of caffeine from the samples increases as the particle size decreases. Superfine grinding is usually accompanied by cell wall rupture, which leads to the accelerated leaching of nutrients [63].

Figure 10c shows that TFAA dissolution rates in BSGTP, OGTP, and CGTP for each extraction time period were closer to each other, while the difference with LJ43 was larger. Presumably, the reason for this is that the LJ43 was not ground, and the cell structure was not destroyed, so the TFAA dissolution was prevented to some extent.

### 3.8. Antioxidant Capacity of BSGTP

**Figure 11:**
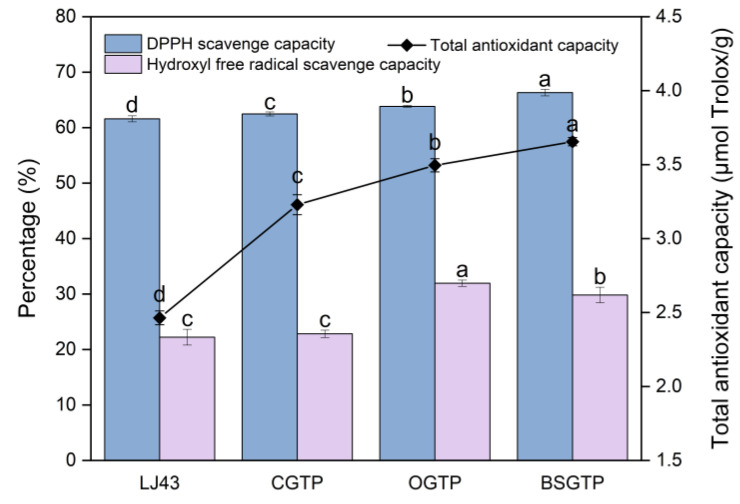
The total antioxidant capacity, DPPH free-radical-scavenging capacity, and hydroxyl free-radical-scavenging capacity of the four sample groups (*p* < 0.05). Different letters a–d: Values in a single indicator with different letters are significantly different (*p* < 0.05).

The total antioxidant capacity, DPPH free-radical-scavenging capacity, and hydroxyl free-radical-scavenging capacity of the four groups of samples are shown in Figure 11.

Based on Figure 11, the total antioxidant capacity and DPPH free-radical-scavenging capacity of the four sample groups are ranked as follows, from lowest to highest: LJ43 < CGTP < OGTP < BSGTP. The total antioxidant capacity and DPPH free-radical-scavenging capacity of BSGTP were significantly higher than the other three groups. The reason for this phenomenon may be that superfine grinding reduces the particle size of tea powder; the powder distribution was more homogeneous, and the specific surface area increased, improving the dissolution of amino acids, caffeine, and other substances in the tea. These substances have antioxidant properties [64,65,66], so the antioxidant capacity of the sample was enhanced. Zhang et al. [67] found that proper superfine grinding can significantly enhance the total antioxidant capacity and DPPH-scavenging capacity of oat bran, and the findings were consistent with the results of this experiment.

Based on Figure 11, the hydroxyl free-radical-scavenging capacities of the four sample groups are ranked as follows, from lowest to highest: LJ43 < CGTP < BSGTP < OGTP. The hydroxyl free-radical-scavenging capacity of BSGTP was significantly lower than that of OGTP and significantly higher than that of LJ43 and CGTP. It is hypothesized that the reason for this phenomenon is that the TPs in green tea have significant hydroxyl-radical-scavenging effects, but the polyphenols were lost during the superfine grinding process due to the continuous heat generation of the equipment [68]. This reduced the hydroxyl free-radical-scavenging capacity of the samples, which is consistent with the results of the TPs content assay in Section 3.6. Hu et al. [30] also indicated that catechin content decreases during the superfine grinding process, which may theoretically reduce antioxidation. In addition, the hydroxyl free-radical-scavenging capacity of BSGTP was only lower than that of OGTP and higher than that of LJ43 and CGTP due to the larger particles in LJ43 and CGTP, resulting in significantly less TPs dissolution compared with BSGTP and OGTP.

## 4. Conclusions

In this study, the effect of the superfine grinding technique on the quality of SGTP was investigated. An AHP–fuzzy comprehensive evaluation system for SGTP was established, and the effects of three factors (grinding time, rotation speed, and ball-to-material ratio) on the main components of SGTP were investigated. The results showed that grinding time, rotation speed, and ball-to-material ratio all significantly affect the main components of SGTP and thus can all affect the quality of SGTP. The ranking of the three factors’ influence on the quality of SGTP is as follows: ball-to-material ratio > grinding time > rotation speed. The SGTP production process was optimized using the RSM. The optimal conditions for preparing SGTP were a grinding time of 5.85 h, a rotation speed of 397 r/min, and a ball-to-material ratio of 9.2:1. Under these conditions, the sample’s AHP–fuzzy comprehensive evaluation score was 83.3519, closely aligning with the predicted value (83.3630) from the model. In addition, compared with green tea powder made using the traditional crushing method, SGTP possesses strong advantages in terms of particle size, the content and dissolution of major components, and antioxidant capacity. The parameters of SGTP are as follows: median particle size (D_50_) of 17 μm, chlorophyll content of 4.77 mg/g, caffeine content of 2.40%, TPs content of 15.97%, TFAA of 2.84%, total antioxidant capacity of 3.65 μmol Trolox/g, DPPH free-radical-scavenging capacity of 66.31%, and hydroxyl free-radical-scavenging capacity of 29.83%.

In the future, it will be necessary to conduct a new optimization study of the preparation process to verify the effect of sensory evaluation (color, taste, aroma, etc.) on the quality of SGTP. In addition, the effects of temperature changes during the superfine grinding process on the main components of tea powder are unknown, and a correlation analysis between different evaluation indexes of tea powder has not been conducted. Therefore, future research should focus on further analyzing the existing results and incorporating sensory evaluation into the evaluation system to find the optimal ball milling process parameters.

## Figures and Tables

**Table 1:** Assignment criteria for indicators in the decision matrix.

Ratio Scale	Meaning
1	Two indicators have equal importance.
2	The former indicator is slightly more important than the latter.
3	The former indicator is more important than the latter.
4	The former indicator is significantly more important than the latter.
5	The former indicator is extremely more important than the latter.

**Table 2:** Fuzzy comprehensive evaluation scoring table.

Item	Scoring Criteria	Score
Chlorophyll	The chlorophyll content is high.	90–99
	The chlorophyll content is relatively high.	80–89
	The chlorophyll content is moderate.	70–79
	The chlorophyll content is relatively low.	60–69
	The chlorophyll content is low.	0–60
Caffeine	The caffeine content is low.	90–99
	The caffeine content is relatively low.	80–89
	The caffeine content is moderate.	70–79
	The caffeine content is relatively high.	60–69
	The caffeine content is high.	0–60
TPs	The TPs content is high.	90–99
	The TPs content is relatively high.	80–89
	The TPs content is moderate.	70–79
	The TPs content is relatively low.	60–69
	The TPs content is low.	0–60
TFAA	The TFAA content is high.	90–99
	The TFAA content is relatively high.	80–89
	The TFAA content is moderate.	70–79
	The TFAA content is relatively low.	60–69
	The TFAA content is low.	0–60

**Table 3:** Gradients of factors and levels.

	Grinding Time (h)	Rotation Speed (r/min)	Ball-to-Material Ratio
1	5	200	6:1
2	6	300	8:1
3	7	400	10:1
4	8	500	12:1
5	9	600	14:1

**Table 4:** Factors and levels of response surface optimization experiment.

Factor		Code Level		
		−1	0	1
A	Grinding time (h)	5	6	7
B	Rotation speed (r/min)	300	400	500
C	Ball-to-material ratio	6:1	9:1	12:1

**Table 5:** Fourth-order judgment matrix.

	Caffeine	Chlorophyll	TPs	TFAA
Caffeine	1	0.5	0.5	0.33
Chlorophyll	2	1	1	0.5
TPs	2	1	1	0.5
TFAA	3	2	2	1

**Table 6:** AHP results.

Item	Eigenvector	Weight Value	Maximum Eigenvalue	CI
Caffeine	0.490	12.252%	4.010	0.003
Chlorophyll	0.909	22.718%		
TPs	0.909	22.718%		
TFAA	1.692	42.312%		

**Table 7:** Summary of consistency test results.

Maximum Eigenvalue	CI	RI	CR	Consistency Test Results
4.010	0.003	0.890	0.004	Pass

**Table 8:** The contents of the major components of SGTP under different grinding time conditions.

Grinding Time (h)	Chlorophyll (mg/g)	Caffeine (%)	TPs (%)	TFAA (%)
5	4.83 ± 0.04 bc	2.38 ± 0.01 c	10.68 ± 0.28 c	2.81 ± 0.02 a
6	5.01 ± 0.11 a	2.41 ± 0.01 b	11.51 ± 0.16 ab	2.76 ± 0.00 b
7	4.93 ± 0.02 ab	2.46 ± 0.01 a	11.81 ± 0.37 a	2.73 ± 0.01 c
8	4.80 ± 0.07 bc	2.41 ± 0.01 b	11.37 ± 0.48 ab	2.71 ± 0.01 c
9	4.73 ± 0.13 c	2.38 ± 0.02 c	10.91 ± 0.26 bc	2.66 ± 0.02 d

Note: Remaining operating conditions: rotation speed of 400 r/min and ball-to-material ratio of 10:1. Data are expressed as the mean standard deviation. Values in the same column with different letters are significantly different (*p* < 0.05).

**Table 9:** The contents of the major components of SGTP under different rotation speed conditions.

Rotation Speed (r/min)	Chlorophyll (mg/g)	Caffeine (%)	TPs (%)	TFAA (%)
200	4.33 ± 0.03 c	2.46 ± 0.00 b	8.47 ± 0.09 e	2.65 ± 0.02 c
300	4.91 ± 0.01 a	2.47 ± 0.01 a	9.92 ± 0.13 d	2.71 ± 0.01 ab
400	4.94 ± 0.01 a	2.45 ± 0.01 bc	10.40 ± 0.04 c	2.72 ± 0.01 a
500	4.85 ± 0.01 b	2.44 ± 0.00 cd	10.58 ± 0.10 b	2.69 ± 0.01 b
600	4.07 ± 0.03 d	2.43 ± 0.00 d	10.96 ± 0.05 a	2.60 ± 0.01 d

Note: Remaining operating conditions: grinding time of 7 h and ball-to-material ratio of 10:1. Data are expressed as the mean plus or minus the standard deviation. Values in the same column with different letters are significantly different (*p* < 0.05).

**Table 10:** The contents of the major components of SGTP under different ball-to-material ratio conditions.

Ball-to-Material Ratio	Chlorophyll (mg/g)	Caffeine (%)	TPs (%)	TFAA (%)
6:1	4.56 ± 0.06 d	2.38 ± 0.01 d	11.11 ± 0.29 b	2.62 ± 0.01 a
8:1	4.77 ± 0.03 c	2.41 ± 0.01 c	11.91 ± 0.14 a	2.63 ± 0.01 a
10:1	5.21 ± 0.06 a	2.44 ± 0.01 b	11.00 ± 0.11 b	2.62 ± 0.01 a
12:1	5.23 ± 0.04 a	2.45 ± 0.01 b	10.83 ± 0.19 b	2.61 ± 0.00 a
14:1	4.97 ± 0.04 b	2.48 ± 0.01 a	9.54 ± 0.10 c	2.58 ± 0.01 b

Note: Remaining operating conditions: grinding time of 7 h and rotation speed of 400 r/min. Data are expressed as the mean standard deviation. Values in the same column with different letters are significantly different (*p* < 0.05).

**Table 11:** Response surface optimization experiment design and results.

Number	A	B	C	Y	
	Grinding Time (h)	Rotation Speed (r/min)	Ball-to-Material Ratio	AHP–Fuzzy Comprehensive Evaluation Score	
				Experimental Score	Model Prediction Score
1	5	500	9	80.3268	79.8540
2	6	400	9	83.3554	83.3496
3	6	300	6	77.5093	76.9448
4	6	400	9	84.0023	83.3496
5	6	400	9	82.9963	83.3496
6	6	300	12	80.1961	79.9112
7	6	400	9	83.1881	83.3496
8	7	400	6	81.3992	81.4909
9	5	400	6	78.2224	78.4102
10	7	400	12	78.1148	77.9269
11	5	400	12	81.9187	81.8269
12	7	500	9	79.0842	78.7075
13	6	500	6	79.4118	79.6967
14	5	300	9	79.0285	79.4052
15	6	500	12	76.0184	76.5830
16	6	400	9	83.2058	83.3496
17	7	300	9	79.2596	79.7324

**Table 13:** Particle size distribution, specific surface area, and cell wall breakage ratio of three sample groups.

	CGTP	OGTP	BSGTP
D_10_ (μm)	12.60 ± 0.55 a	8.09 ± 0.43 b	4.09 ± 0.11 c
D_50_ (μm)	168 ± 1.36 a	92 ± 0.97 b	17 ± 0.36 c
D_90_ (μm)	381 ± 2.70 a	223 ±1.88 b	43.7 ± 0.53 c
D_(3, 2)_ (μm)	33.3 ± 0.92 a	22.0 ± 0.40 b	9.75 ± 0.25 c
D_(4, 3)_ (μm)	183 ± 1.53 a	103 ± 0.58 b	21.2 ± 0.71 c
SPAN (μm)	2.186 ± 0.004 b	2.336 ± 0.001 a	2.334 ± 0.002 a
Specific Surface Area (m^2^/kg)	180.4 ± 1.85 c	272.6 ± 1.16 b	615.5 ± 2.35 a
Φ (%)	16.815 ± 0.13 c	29.193 ± 0.27 b	93.019 ± 0.63 a

Note: D_10_, the diameter of 10% of particles below this value; D_50_, the median particle size, the diameter of 50% of particles below this value; D_90_, the diameter of 90% of particles below this value; D_(3, 2)_, the surface weighed mean diameter; D_(4, 3)_, the volume-weighted mean diameter; SPAN, the distribution width; Φ, the cell wall breakage ratio. Values in the same raw with different letters are significantly different (*p* < 0.05).

## Data Availability

The original contributions presented in the study are included in the article/Supplementary Materials, further inquiries can be directed to the corresponding author.

## Supplementary Materials

The following supporting information can be downloaded at https://www.mdpi.com/article/10.3390/foods14071283/s1, Figure S1. HPLC chromatograms of the caffeine standard and BSGTP: (a) caffeine standard, (b) BSGTP.
