# Supplementary material for: Optimization of the Ball Milling Process for Producing Superfine Green Tea Powder: An Analytic Hierarchy Process–Fuzzy Comprehensive Evaluation Approach
Source: Foods. 2025 Apr 7;14(7):1283. doi: 10.3390/foods14071283 (PMC11989055; doi:10.3390/foods14071283)
Supplement: Supplementary file 1 [file foods-14-01283-s001.zip › foods-3498278-supplementary.pdf]

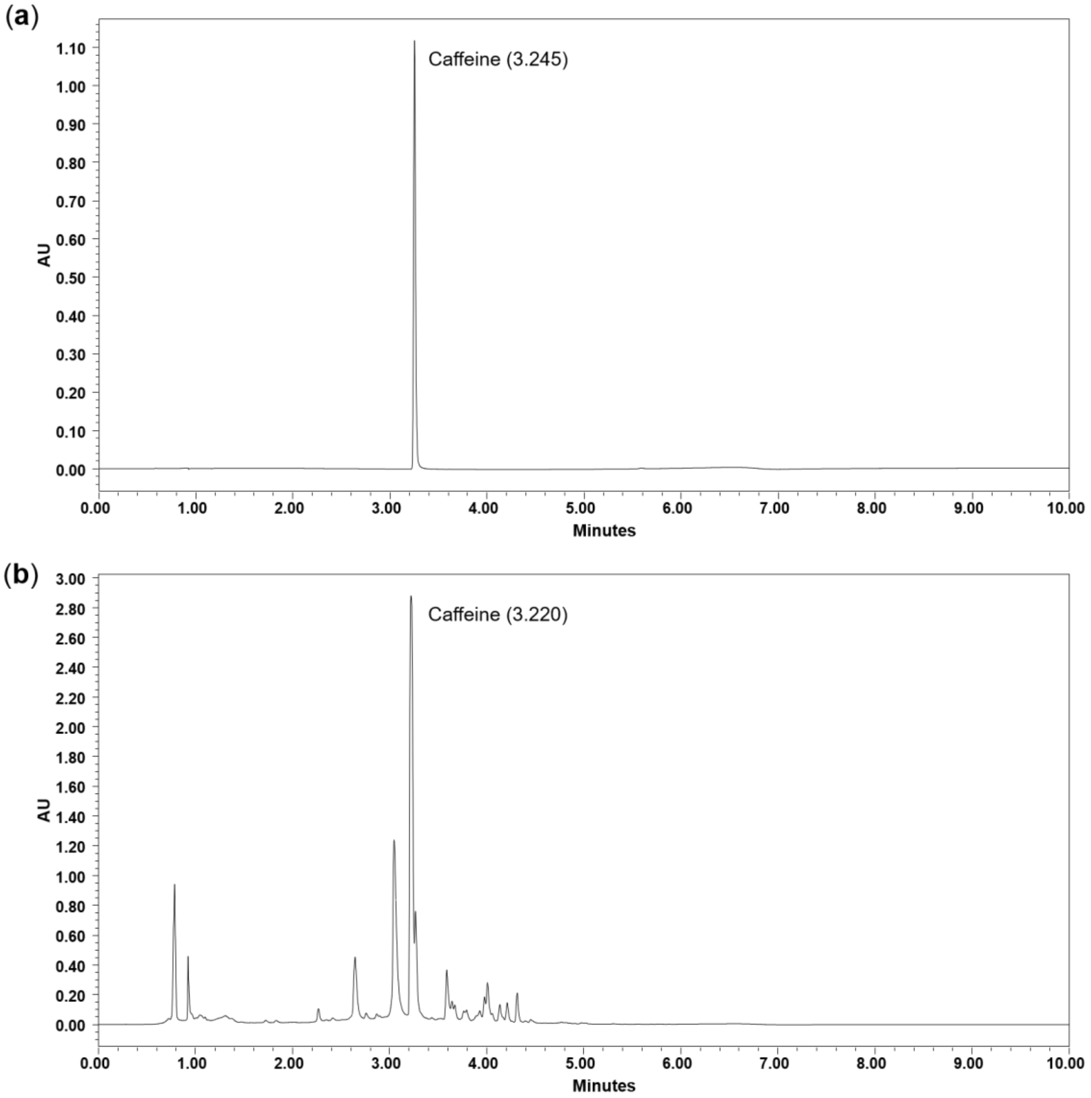

Figure S1. HPLC chromatograms of the caffeine standard and BSGTP. (a) caffeine standard, (b) BSGTP.

Figure S1(a) and Figure S1(b) show that the retention time was 3.245 min for the caffeine standard and 3.220 min for BSGTP. The retention times of both were close to each other, indicating that the retention behavior of caffeine in the samples was consistent with that of the standard.
